# Is Pressurized Intraperitoneal Aerosolized Chemotherapy (PIPAC) Effective in Ovarian Cancer With Peritoneal Metastasis?

**DOI:** 10.7759/cureus.27837

**Published:** 2022-08-09

**Authors:** Amad Mohammad, Mosab Hor, Ahmed M Baradeiya, Hodan Qasim, Mohamed Nasr

**Affiliations:** 1 Hematology and Oncology, Saint James School of Medicine (SJSM), New York, USA; 2 Ophthalmology, Palestinian Medical Council, Ramallah, PSE; 3 Research, Fresno Clinical Research Center, Fresno, USA; 4 Internal Medicine, Alfaisal University, Riyadh, SAU; 5 General Internal Medicine, Mansoura General Hospital, Mansoura, EGY

**Keywords:** peritoneal metastasis, hipec, peritoneal carcinomatosis, recurrent ovarian cancer, peritoneal cancer, ovarian metastasis, intraperitoneal (ip) chemotherapy, chemotherapy, ovarian cancer, pipac

## Abstract

Ovarian cancer is one of the most common causes of mortality in women and is frequently diagnosed at an advanced stage. Ovarian cancer has a high recurrence rate, with most cases being peritoneal metastasis. The standard treatment of peritoneal metastasis is systemic chemotherapy, but naturally, the peritoneum is poorly vascularized, making this standard of treatment frequently ineffective. Hence, pressurized intraperitoneal aerosol chemotherapy (PIPAC) introduced a new type of intraperitoneal chemotherapy (IPC) in November 2011. Positive feedback on its feasibility, tolerance, and efficacy has encouraged medical communities worldwide to adopt PIPAC as a new drug delivery technique. This study's objective is to review previously conducted research on the efficacy of PIPAC treatment for peritoneal metastasis from ovarian cancer.

## Introduction and background

The most common cause of mortality in women with female reproductive cancers is ovarian cancer, and in general, it is the fifth leading cause of death in women [1]. Usually diagnosed at an advanced stage, ovarian cancer recurrence develops in 60%-85% of patients within five years post-primary treatment, with 80% of all cases recurring with peritoneal metastasis [2,3]. Systemic chemotherapy is the standard of care in women with recurrent ovarian cancer, and frequently, peritoneal metastasis is refractory to this type of treatment [2,4]. Treatment resistance in peritoneal metastasis attributes to numerous reasons. One theory is that the plasma-peritoneal barrier prevents and limits systemic chemotherapy from reaching the peritoneal cancer nodules, causing low response rates compared to metastatic spread in other organs (e.g., liver and lung) [5]. Most chemotherapy (95%-98%) administered intravenously bypasses the peritoneum and causes systemic adverse effects [6].

In November 2011, a minimally invasive intraperitoneal drug therapy delivery called pressurized intraperitoneal aerosol chemotherapy (PIPAC) was introduced for patients with primary peritoneal carcinomatosis or peritoneal metastases [4,7]. Intraperitoneal chemotherapy (IPC) is an alternative treatment to systemic chemotherapy in patients with recurrent ovarian cancer [8]. Intraperitoneal administration of chemotherapy allows the drugs to directly target the cancer cells within the peritoneal cavity [8]. Intraperitoneal chemotherapy as adjuvant therapy is also proven beneficial in women with ovarian cancer after primary debulking surgery [8]. Randomized clinical trials and meta-analyses have shown that adjuvant IPC with cisplatin and paclitaxel combined with intravenous chemotherapy halts cancer progression and improves survival altogether [8].

Medical communities worldwide are increasingly adopting PIPAC as a new drug delivery technique due to the positive feedback from clinical trials [4]. As an alternative form of IPC, PIPAC repeatedly applies aerosolized chemotherapeutic drugs under pressure via a laparoscopic surgical procedure [8]. Based on observation, this approach of using pressure via laparoscopy shows enhanced drug uptake by the tumor [8]. Furthermore, administering chemotherapeutic agents as an aerosol may enhance more surface area coverage of the peritoneum and can increase drug penetration [8]. Also, PIPAC can be applied more frequently than other intraperitoneal chemotherapeutic treatments, increasing its goal of local control of peritoneal carcinomatosis and physiological homeostasis [8].

Since intraperitoneal chemotherapy is an experimental approach for peritoneal metastasis in ovarian cancer and other intra-abdominal cancers (e.g., gastric cancer and colon cancer), this mode of therapy has a very high potential to improve survival in these types of cancer patients [2]. This review article will discuss the effectiveness of PIPAC in patients with recurrent ovarian cancer with peritoneal metastasis.

Methods and search strategy

Extensive research was conducted using the following keywords to recognize the studies analyzing and assessing PIPAC treatment for peritoneal metastasis in patients with ovarian cancer using PubMed, Google Scholar, and ScienceDirect databases: PIPAC, ovarian cancer, chemotherapy, intraperitoneal (IP) chemotherapy, ovarian metastasis, peritoneal cancer, recurrent ovarian cancer, peritoneal carcinomatosis, HIPEC, and peritoneal metastasis. Apart from the study's primary aim, ovarian cancer with peritoneal metastasis community development, pathophysiology, epidemiology, treatment, and management alternatives are included in great detail in this study. All the articles considered were chosen without the restriction of time of publication or study type, i.e., traditional reviews, systematic reviews, clinical trials, case-control studies, and cohort studies. Studies were not refined based on age and ethnicity. There were no demographical limitations in the search. All the articles chosen were in the English language. Data collection is from inception to May 2022.

## Review

This section will discuss the epidemiology of ovarian cancer and peritoneal metastasis along with the pathophysiology of the disease process with an emphasis on PIPAC treatment. The discussion will also include alternative treatments and limitations of PIPAC with its efficacy. Various clinical trials demonstrating the use of PIPAC in ovarian cancer with peritoneal metastasis are also summarized.

Epidemiology

Every year, 225,500 patients worldwide are diagnosed with ovarian cancer [9]. In the past 10 years, mortality has barely improved, with the mean age of diagnosis between 55 and 65 years [9,10]. The incidence steadily increases with advanced age and family history of ovarian and breast cancer, which are also significant risk factors [10]. Due to the lack of symptoms, diagnoses of more than 75% of women are at advanced stages, and more than 60% have metastasized to other organs at the time of diagnosis, which explains the high mortality rates in ovarian cancer [10].

Ovarian cancer usually remains local and metastasizes by direct extension to neighboring organs, such as the bladder (17%) and colon, or by the detachment of cancer cells that metastasize into every intraperitoneal structure by transperitoneal dissemination [10]. The intraperitoneal structures that are commonly affected are the peritoneum and omentum (86%), bowel (50%), and spleen (20%) [10]. Vascular metastasis spread is not typical in ovarian cancer (16%), with the most common sites for vascular metastasis being the pleura (33%), liver (26%), and lung (15%) [10].

Secondary peritoneal cancer is due to metastasis and is the most common cancer in the peritoneal cavity [11]. Metastasis that arises from ovarian, gastric, and colorectal cancers are all associated with high rates of recurrence and mortality. These three cancers are the most common cause of peritoneal metastasis [11].

Pathophysiology

**Figure 1:**
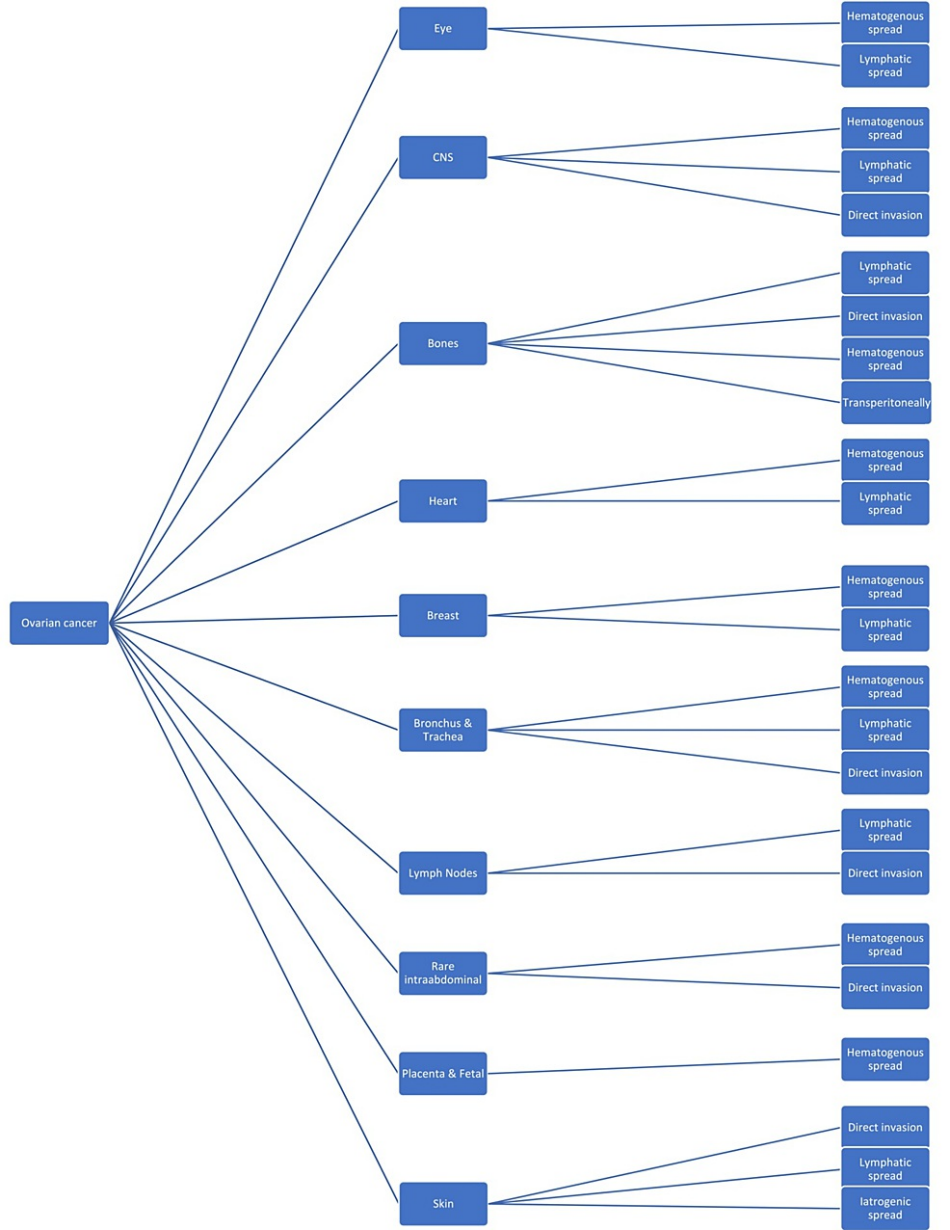
Distant metastatic sites of ovarian cancer with their transmission routes

The exact etiology of ovarian cancer with peritoneal metastasis is not entirely understood. However, studies have proved that factors such as genetic predisposition, nulliparity, and benign inflammatory diseases all give rise to ovarian cancer [10]. Metastasis is a common finding in patients with ovarian cancer, with the most common locations being the peritoneum, abdomen, and pelvic organs [10]. These metastasis sites are not entirely random and spread by direct invasion; however, other routes such as lymphatic and hematogenous vessels also play a significant role, especially for the less common metastatic sites [10]. The least common metastatic sites are the skin, bone, central nervous system (CNS), eyes, breast, bronchus and trachea, heart and pericardium, rare lymph nodes, and scarce intra-abdominal sites [10]. Figure 1 outlines these rare distant metastatic sites' transmission routes [10].

Ovarian cancer metastases can occur via three significant dissemination routes: transcoelomic (metastatic spread across the peritoneal cavity), lymphatic (metastatic spread through the lymph system), and hematogenous (metastatic spread through the blood) [12]. The transcoelomic route is the most common route of dissemination and is responsible for the peritoneal metastases observed in 70% of patients and is associated with increased mortality and morbidity due to their ability to affect surrounding organs [12]. Moreover, transcoelomic metastases are also associated with malignant ascites and are commonly present in advanced stages of ovarian cancer [12]. Ascites can lead to multiple problems including, but not limited to, intestinal function impairment due to external obstruction and restrictive breathing [13]. The pathophysiology of ascites is multifactorial, but a few mechanisms proposed include lymphatic obstruction and increased vascular permeability [12]. A correlation appears between the number of metastatic lymph nodes and the development of ascites [12].

The role of the peritoneum in metastatic tumor spread has been significantly researched. It is found that peritoneal cancers are often discovered incidentally and are responsible for the increased fatality in patients with ovarian cancer with peritoneal metastasis [11]. The predominant role of peritoneal cells is regulating intraperitoneal homeostasis of the abdominal cavity by exchanging molecules and fluids [14]. In addition, the peritoneum plays an essential role in inflammatory responses, antigen presentation, fibrosis and fibrinolysis, tissue repair, and the development of peritoneal metastases [14]. The carcinogenesis of peritoneal cancers can be further explained by the "seed and soil theory" by Stephen Paget [15]. It describes how a malignant tumor gives up cells (seeds) that travel in all directions but can only survive and multiply at tumor-accepting locations (soil). It explains the inclination of colorectal, ovarian, and gastric tumor cells for the peritoneum [15].

Clinical trials of PIPAC treatment in patients with peritoneal cancer

We identified four studies describing the experimental evidence, methods, outcomes, and clinical applications of PIPAC in women with gynecologic malignancies. Of these four studies, one is experimental, assessing operational safety, occupational exposure, and room contamination [16]. The other three studies reported clinical outcomes in women with ovarian and peritoneal cancers [17-19]. Table 1 lists all studies detailing study characteristics such as the number of patients and outcomes.

**Table 1:** Clinical trials of PIPAC treatment in patients with peritoneal cancer

Author	Year	Oncology dx	Number of patients	Outcomes
Solaß et al. [16]	2013	Not available	2	Operational safety, occupational exposure, and room contamination
Solaß et al. [17]	2012	Gastric, appendiceal, and ovarian cancer	3	Feasibility, pharmacokinetics, and histological response
Tempfer et al. [18]	2014	Ovarian cancer	18	Radiological, histological response, and local and systemic toxicity
Blanco et al. [19]	2013	Peritoneal cancer	3	Acute and cumulative hepatic and renal toxicity

Treatment

The best treatment approach for peritoneal cancer from ovarian cancer metastasis is multimodal [11]. A combination of surgery, chemotherapy, and targeted therapy is the gold standard of treatment [11]. Most ovarian carcinomas are managed the same way as the serious ones, which involve hysterectomy, bilateral salpingo-oophorectomy, and omentectomy, followed by chemotherapy [11]. Platinum-based chemotherapy is incredibly advantageous; however, many platinum-resistant tumors have proven unresponsive, and multimodal therapy is beneficial in these cases [11]. A phase III clinical trial has shown that intraperitoneal chemotherapy is superior to intravenous therapy in overall survival (60 months versus 50 months) [11]. Debulking surgery, also called cytoreductive surgery (CRS), is performed with chemotherapy with wide excision of the tumor with <2 cm residual nodules and has also shown optimal results [11]. At the same time, salvage chemotherapy is used in tumor recurrence and includes doxorubicin, methotrexate, paclitaxel, and 5-fluorouracil [11].

Cytoreductive Surgery (CRS) and Hypothermic Intraperitoneal Chemotherapy (HIPEC)

CRS is the procedure that removes all the tumor nodules from the parietal and visceral peritoneal layers [11]. The procedure requires a skillful surgeon with excellent technical skills and conduction of perfect hemostasis [11]. Complete and successful cytoreduction occurs when no visible tumor nodules remain after the surgery [20]. Postoperative complications include and are not limited to veno-thrombotic events, operative site abscess, anastomotic leaks, fistula, and long-term intensive care stay [11].

HIPEC involves pumping chemotherapy drugs at a temperature higher than the average body temperature into the peritoneal cavity for two hours [11]. Hyperthermia impairs DNA repair, induces apoptosis, inhibits angiogenesis, and promotes protein denaturation, causing the cancer cells to die at 104°F while the healthy cells survive until 111°F [11]. Also, laparoscopic HIPEC is very effective in refractory malignant ascites, significantly lowering morbidity and mortality of patients with peritoneal carcinomatosis [11]. Data also shows that in patients with no macroscopic residual disease, CRS + HIPEC increases survival rates [21]. Moreover, cytoreductive surgery combined with hyperthermic intraperitoneal chemotherapy (CRS + HIPEC) has also shown to be very effective in prolonging survival in selected patients with colorectal cancer peritoneal carcinomatosis [22]. However, unfortunately, the adverse effects of HIPEC include neutropenia, spontaneous bowel perforations, electrolyte imbalance, acute renal failure, and bleeding diathesis [11].

Pressurized Intraperitoneal Aerosol Chemotherapy (PIPAC)

PIPAC is a new approach to administering intraperitoneal chemotherapy drugs minimally invasively [11]. The procedure is conducted in patients when CRS + HIPEC is not an indication of a considerable tumor load or in patients with significant persistent ascites [11]. PIPAC drug delivery can be repeated safely without systemic chemotherapy effects and with decreased chemical bowel perforations in contrast to HIPEC [11]. Notably, PIPAC shows minimal hepatic and renal toxicities [23]. However, the procedure has contraindication in patients with biliary or small bowel obstructions and in patients with extra-abdominal metastasis [11]. The classical side effects from systemic chemotherapy, such as mucositis, nausea/vomiting, diarrhea, paresthesia, cutaneous symptoms, and alopecia, are also not reported by treated patients [24].

Technique and Efficacy of PIPAC

PIPAC is a type of intraperitoneal chemotherapy (IPC) to target cancer cells via laparoscopy directly, and the technique performed is as follows [8]. A standard CO2 pneumoperitoneum (capnoperitoneum) is established; about 12 mmHg of insufflation and two access balloon trocars are inserted through the abdominal wall [24]. Peritoneal biopsies are taken in all four quadrants for histological staging and the determination of the peritoneal cancer index (PCI) [25]. The biopsied nodules are then documented and removed if ascites or mucus volumes are found [24]. Next, a nebulizer or aerosolizer connects to an intravenous high pressurized aerosol containing chemotherapy solution (e.g., cisplatin and doxorubicin) applied via the aerosolizer and injector [24]. The injection is remote-controlled, with nobody remaining in the room to minimize occupational exposure during the application [25]. The therapeutic capnoperitoneum is maintained for 30 minutes at an average body temperature (37°C) [25]. Once the 30 minutes is over, the chemotherapy aerosol is exsufflated into the air waste system of the hospital [25]. Finally, the trocars are retracted, and the laparoscopic procedure is complete with no drainage of the abdomen necessary [25].

More clinical trials are required to test for its efficacy and usage, but in recent years, data regarding PIPAC with low-dose cisplatin and doxorubicin or oxaliplatin shows promising results [11,26]. Studies on peritoneal carcinomatosis from intestinal, appendiceal, gastric, and ovarian cancers have emphasized its safety and better tolerability, with a median survival rate of 15.7 months [11]. Using a strict response evaluation process, PIPAC has shown to statistically induce histological regression in patients with peritoneal metastasis with an objective observation of tumor response in most patients [19]. The most striking result from the studies is the quality of life (QoL) under PIPAC treatment [24]. In one study, the QoL was assessed before starting PIPAC and over three months during the treatment with no change in QoL stabilization [24]. However, the disadvantage of PIPAC is that adhesions secondary to surgery create obstacles to aerosol diffusion [11]. Hence, this is not a good option in patients with recurrence after CRS [11]. It is essential to mention that tumor response, clinical response, and QoL showed significant improvement in a bidirectional treatment, meaning PIPAC with the addition of systemic chemotherapy [11].

Limitations of the study and occupational safety of PIPAC

This study has several limitations. Chemotherapy as an aerosol might cause an increased risk of exposure to healthcare workers compared to other administration routes [16]. However, occupational safety is assessed in operating rooms with laminar airflow [3]. Trocars are secured with an airtight intra-abdominal balloon to prevent leakage of agents, and the gas in the abdomen is disposed of through the hospital's waste air system at the end of the procedure [3]. The risk of skin contamination is low due to the closed system of PIPAC [3]. More studies are required to investigate the occupational safety of other chemotherapeutic agents [3].

Also, in the absence of controlled results comparing PIPAC versus standard of care, PIPAC-directed treatment has not yet reached the level of evidence for full recognition within the medical community [27]. More research on strategies and tools for objective response evaluation is needed, and the present data should be confirmed in more extensive PIPAC studies [28]. However, with time and more clinical studies, accepting PIPAC is inevitable.

## Conclusions

Ovarian cancer is the fifth leading cause of death in women, and systemic chemotherapy is commonly refractory to peritoneal metastasis. The global medical community is adopting the relatively new treatment of pressurized intraperitoneal aerosolized chemotherapy (PIPAC) for peritoneal cancer. Experimental and clinical data have shown that PIPAC is safe and effective for treating peritoneal cancer from ovarian cancer metastasis. Studies have also shown that the quality of life in treated patients is immensely more positive than the traditional standard of care from systemic chemotherapy. Patients do not report the classical side effects of systemic chemotherapy, such as mucositis, nausea/vomiting, diarrhea, paranesthesia, and alopecia. Nevertheless, with the absence of controlled results comparing PIPAC versus standard of care, PIPAC-directed treatment has not yet reached the level of evidence for full recognition. Suggestions for future trials include, but are not limited to, strategies and tools for objective response evaluation, but with time and more clinical research, the acceptance of PIPAC is inevitable.
